# A New Low Cost Wide-Field Illumination Method for Photooxidation of Intracellular Fluorescent Markers

**DOI:** 10.1371/journal.pone.0056512

**Published:** 2013-02-18

**Authors:** Manoel da Silva Filho, Daniel Valle Vasconcelos Santos, Kauê Machado Costa

**Affiliations:** Institute of Biological Sciences, Federal University of Pará, Belém, Brazil; Federal University of Rio de Janeiro, Brazil

## Abstract

Analyzing cell morphology is crucial in the fields of cell biology and neuroscience. One of the main methods for evaluating cell morphology is by using intracellular fluorescent markers, including various commercially available dyes and genetically encoded fluorescent proteins. These markers can be used as free radical sources in photooxidation reactions, which in the presence of diaminobenzidine (DAB) forms an opaque and electron-dense precipitate that remains localized within the cellular and organelle membranes. This method confers many methodological advantages for the investigator, including absence of photo-bleaching, high visual contrast and the possibility of correlating optical imaging with electron microscopy. However, current photooxidation techniques require the continuous use of fluorescent or confocal microscopes, which wastes valuable mercury lamp lifetime and limits the conversion process to a few cells at a time. We developed a low cost optical apparatus for performing photooxidation reactions and propose a new procedure that solves these methodological restrictions. Our “photooxidizer” consists of a high power light emitting diode (LED) associated with a custom aluminum and acrylic case and a microchip-controlled current source. We demonstrate the efficacy of our method by converting intracellular DiI in samples of developing rat neocortex and post-mortem human retina. DiI crystals were inserted in the tissue and allowed to diffuse for 20 days. The samples were then processed with the new photooxidation technique and analyzed under optical microscopy. The results show that our protocols can unveil the fine morphology of neurons in detail. Cellular structures such as axons, dendrites and spine-like appendages were well defined. In addition to its low cost, simplicity and reliability, our method precludes the use of microscope lamps for photooxidation and allows the processing of many labeled cells simultaneously in relatively large tissue samples with high efficacy.

## Introduction

Analyzing cell morphology is a crucial aspect of cell biology and neuroscience. Relationships between form and function define physiological processes in health and disease. One of the main methods for evaluating cell morphology is through the use of intracellular fluorescent markers, including various commercially available dyes, fluorochrome labeled antibodies and genetically encoded fluorescent proteins, such as green fluorescent protein (GFP). These fluorophores can be visualized directly under fluorescent, confocal or multi-photon microscopy, allowing the investigator to directly observe the targeted cell, including cellular substructures. Fluorescent markers can also be used for conventional bright field optical microscopy and transmission electron microscopy through a process called fluorescent photooxidation. This technique, initially developed by Maranto [1], is based on photoconverting certain intracellular markers in order to promote the oxidation of 3,3 ′-diaminobenzidine tetrahydrochloride (DAB) with high spatial precision and acuity. This is possible due to the fact that most organic fluorophores release singlet oxygen molecules when illuminated. In the presence of a DAB solution, the reactive free radicals oxidize this organic compound, forming an opaque, electron-dense and osmiophilic brown polymer [1], [2]. Because singlet oxygen molecules are only released where there are fluorescent markers and exclusively upon illumination with an adequate light wavelength, the investigator has sufficient control of the temporal and spatial parameters of the photooxidation reaction, thus being capable of regulating the structural specificity and intensity of the subsequent DAB staining. In addition, the resulting staining does not suffer from photobleaching and can be applied to correlative light and transmission electron microscopy [1], [2].

While photooxidation was initially executed with Lucifer Yellow, it has been subsequently demonstrated in a variety of cell types that other fluorescent dyes can be applied for the same purpose, provided they are exposed to light with a spectrum that corresponds to their excitation wavelength [2]. Examples of fluorophores that have been used for fluorescent photooxidation include DiI, DiO, Fast Blue, Fluorogold and rhodamine labeled microspheres [3]–[8]. These dyes have the advantage of providing high quality staining of neuronal processes, and have thus been extensively used for neurite mapping studies [1], [3], [7], [9], [10]. Fluorescent secondary antibodies for immunohistochemistry and specific histochemical stains, such as DAPI and Eosin, can be used for targeted photooxidation of specific proteins and cellular structures [4], [11], [12]. Methods for photooxidation of fluorescent genetically encoded markers have also been developed in the last decade. Fluorescent photooxidation was initially applied to enhanced green fluorescent protein (EGFP) by Grabenbauer et al. [13], which can be used for visualizing specific organelles and proteins with a high signal to noise ratio. More recently, Shu et al. [14] developed a specific genetically encoded marker for fluorescent photooxidation. The novel marker, named “MiniSOG”, was developed for optimal photooxidation and targeted correlative light and electron microscopy in intact tissue.

However, all photooxidation techniques developed so far are dependent on the optical apparatus of fluorescent or confocal microscopes to trigger fluorophore photoconversion. In current photooxidation protocols, the investigator must irradiate a specific region of the sample, as defined by the objective lens of the microscope. The necessary illumination time can vary from a few minutes to up to several hours depending on the used fluorophore, the cellular substrate to be analyzed and the objective of the study [2], [3], [15]. This process wastes valuable mercury lamp lifetime and takes up microscope user time, which is commonly a scarce resource in life science laboratories. We describe here the design and construction of a novel low cost optical apparatus that uses a high power light emitting diode (LED) to irradiate fluorescent markers for controlled DAB photooxidation. We show that our new “photooxidizer” is capable of reliably inducing photooxidation reactions, resulting in high quality staining of cells in nervous tissue with much higher efficiency than currently available methods.

## Materials and Methods

### Ethics statement

All animal experiments were carried out in accordance with the National Institute of Health Guide for the Care and Use of Laboratory Animals (NIH Publications No. 80-23, revised 1996), and were approved by the Ethical Research Committee for Animal experiments of the Institute of Biological Sciences, Federal University of Pará (protocol N°: 3988/2011). Animals were obtained from the Animal Service of the Institute of Biological Sciences of the Federal University of Pará. Samples of fixed post-mortem human retinas were obtained from the Ophir Loyola Hospital's Eye Bank (Belém, PA, Brazil). Written statements from family members of the deceased subjects is a prerequisite for tissue storage in this bank. All procedures with post-mortem human tissue were approved by the Committee on Research Ethics of the Federal University of Pará (protocol N°: 2012/20468).

### Photooxidizer apparatus

**Figure 1:**
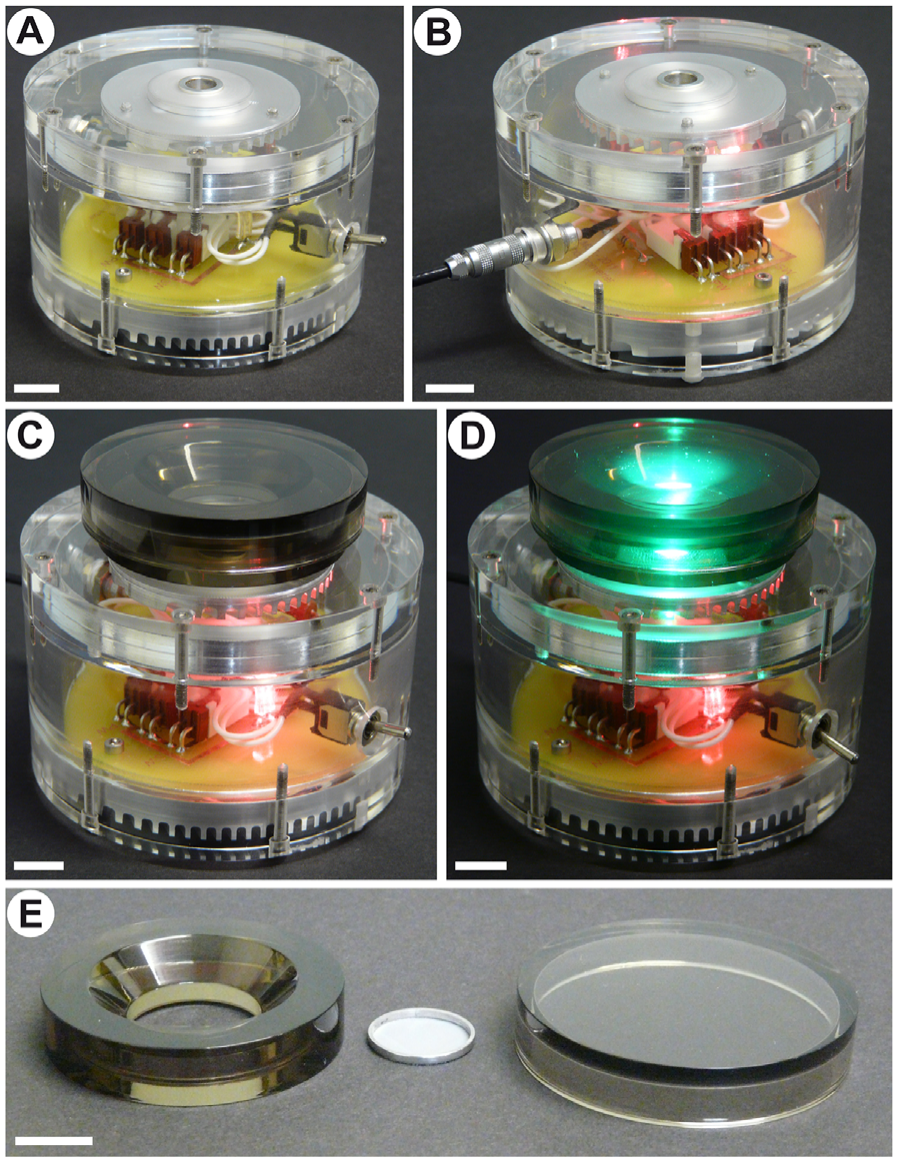
Photooxidizer apparatus and tissue chamber. Scale bars correspond to 10 mm. A: Front view of the apparatus in an offline configuration. B: Rear view of the apparatus. The machine is connected to an outlet, but the switch is turned off. C: View of the photooxidizer with the connected tissue chamber. D: View of the photooxidizer with the connected tissue chamber while the apparatus is turned on. Note that the chamber fits over the LED light beam. E: Disassembled tissue chamber, including the acrylic ring, the glass lid and the aluminum ring with nylon mesh used for holding the tissue in place.

**Figure 2:**
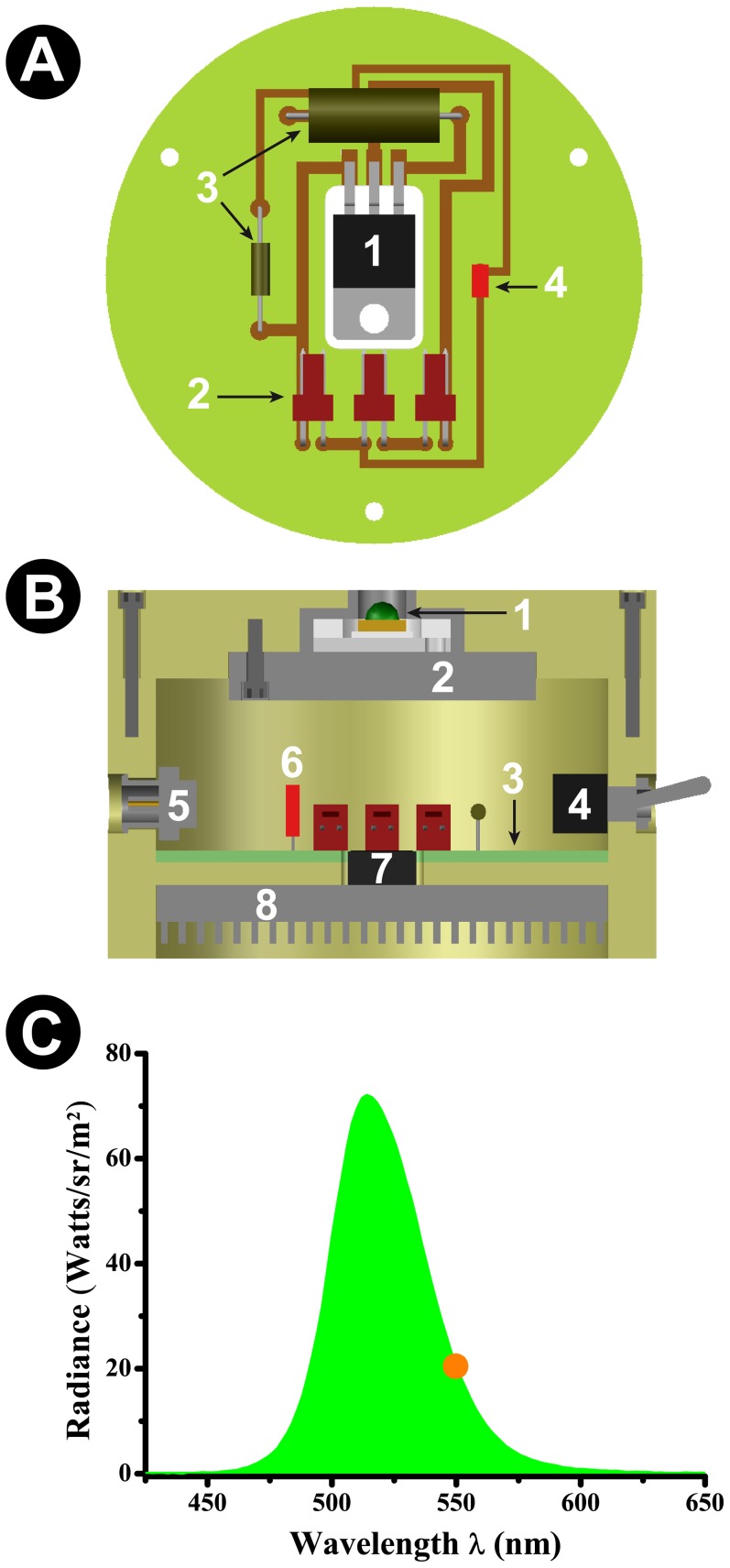
Schematics and function of the photooxidizer. A: Schematic drawing of the PCB. 1: Voltage regulator microchip; 2: Input/output connectors; 3: Resistors; 4: Red low power LED (indicates power feed). B: Schematic drawing of the internal components of the photooxidizer. This panel represents a vertical transection of the apparatus. 1: Green high power LED (promotes photooxidation); 2: LED chamber heat sink; 3: PCB; 4:On/off switch; 5: Power feed connector; 6: Red low power LED (indicates power feed); 7: Voltage regulator microchip; 8: PCB heat sink. C: Radiance spectrum of the green high power LED used for DiI photoconversion. The orange dot represents the peak absorbance wavelength of DiI, confirming the appropriateness of the used LED.

For our apparatus, we used a high power LED (XR7090GRN, Cree) powered by a voltage regulator microchip (LM 317, Texas Instruments). The nominal current for this LED is 350 mA for a 3.6 Ω resistor, but we chose to use an associated resistor of 3.9 Ω, resulting in a current of 320 mA to the LED. This difference does not affect the photooxidation process, but increases the LED's lifespan substantially. A red low power LED indicates if the circuit is being fed (Figure 1, Panels B–D). The complete circuit was mounted in a circular printed circuit board (Figures 1 and 2). The spectral emission of the high power LED was measured with a spectroradiometer (SpectraScan 705; Photo Research, USA) and is represented graphically in Figure 2, Panel C.

The high power LED is fixed within a cylindrical aluminum chamber. The LED inner circuit is isolated from the aluminum chamber by a *Teflon®* ring. A heat sink (an aluminum grooved plate) is screwed to the underside of the LED chamber and thermal paste is added to allow a more effective heat transfer. The LED chamber and the PCB are fixed within a cylindrical transparent acrylic case. The case is equipped with an external power connector (NIM-CAMAC 00.250 series - fixed socket, Lemo, Switzerland) and an on/off switch (Spst micromini toggle switch, RadioShack, USA) that controls the high power LED's function. At the underside of the acrylic case an aluminum heat sink is added, necessary for dissipating the 1.8 W of power generated by the PCB's circuit. For detailed information on the circuit board configuration and photooxidizer assembly see Supplemental Information (Tables S1 and S2; File S1).

### Tissue chamber

During the photooxidation reaction, tissue samples are placed within a custom-made tissue chamber (Figure 1E). This piece consists of a translucent acrylic ring with a glass coverslip glued to its underside. The inner hole of the ring has a conical recess, in order to better accommodate the objective lens of a microscope. This chamber allows the investigator to transfer the samples from the photooxidizer to the microscope and back easily, thus facilitating the monitoring of the photooxidation reaction, a critical factor for obtaining high quality images. In order to avoid light dispersion, the chamber has a tightly fit translucent acrylic lid. Tissue samples are held in place by a stainless steel ring with an internal nylon mesh (Figure 1E).

**Figure 3:**
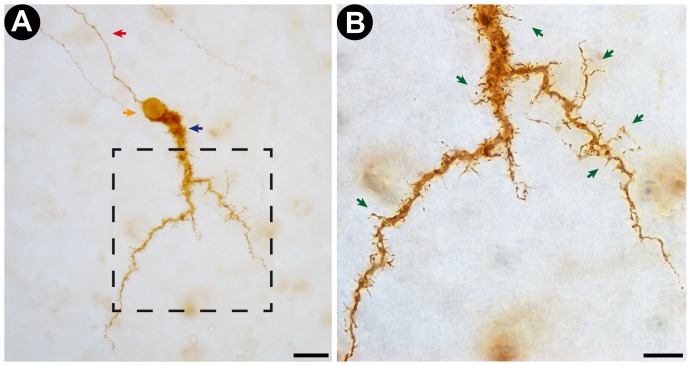
Representative photomicrograph of a Cajal-Retzius cell stained by the new photooxidation method. A: Low magnification (40× objective lens) photomicrograph of the DAB-stained Cajal-Retzius Cell obtained from a transverse section of developing rat neocortex. Note that the cell is stained over its entire volume, with no variations in staining intensity. The red arrow points at the cell's axon. The blue arrow indicates the large horizontal dendrite that defines the Cajal-Retzius cell type. The orange arrow points at the cell's soma Scale bar corresponds to 20 µm. B: High magnification (100× objective lens) photomicrograph of the insert indicated in A (dotted line square). Note the successful staining of dendritic shafts and spine-like appendages (green arrows), highlighting the high degree of detail and acuity of the staining. Scale bar corresponds to 10 µm.

**Figure 4:**
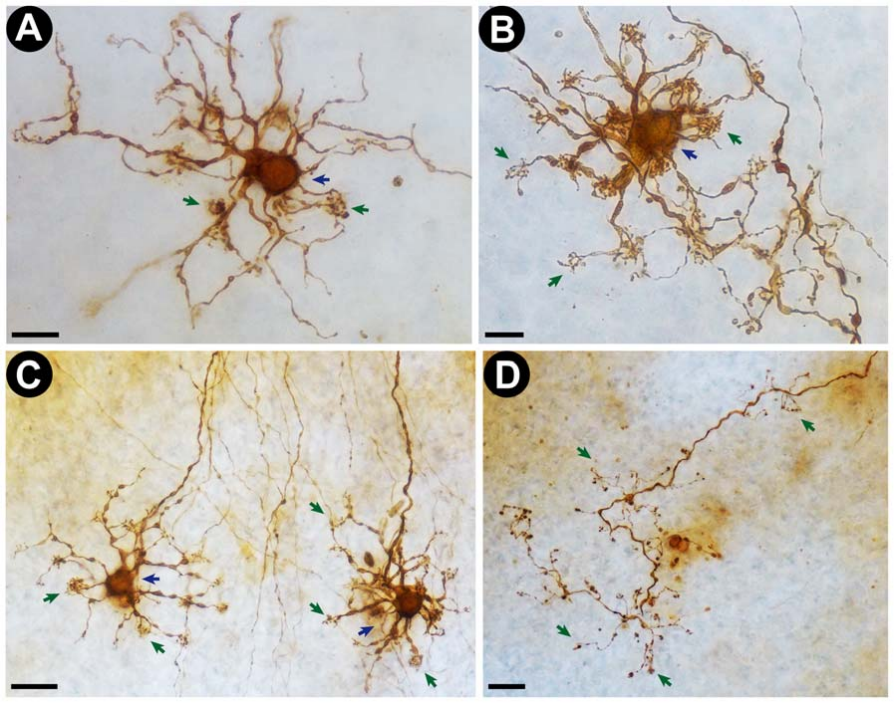
Representative photomicrographs of human retinal neurons and axons stained by the new photooxidation method. Cells were processed and observed in intact post-mortem human retinas. A and B: High magnification (100× objective lens) photomicrographs of DAB-stained putative horizontal retinal neurons. Note the clear delineation of the cell body (blue arrows) and dendritic tufts (green arrows). Scale bars correspond to 10 µm. C: Low magnification (40× objective lens) photomicrograph of two DAB-stained retinal cells (putatively horizontal cells). Scale bar corresponds to 20 µm. D: Low magnification (40× objective lens) photomicrograph of a DAB stained axon terminal in a sample of human retina. Note that even fine structures, such as axonal knobs (arrows), are clearly stained. Scale bar corresponds to 20 µm.

**Figure 5:**
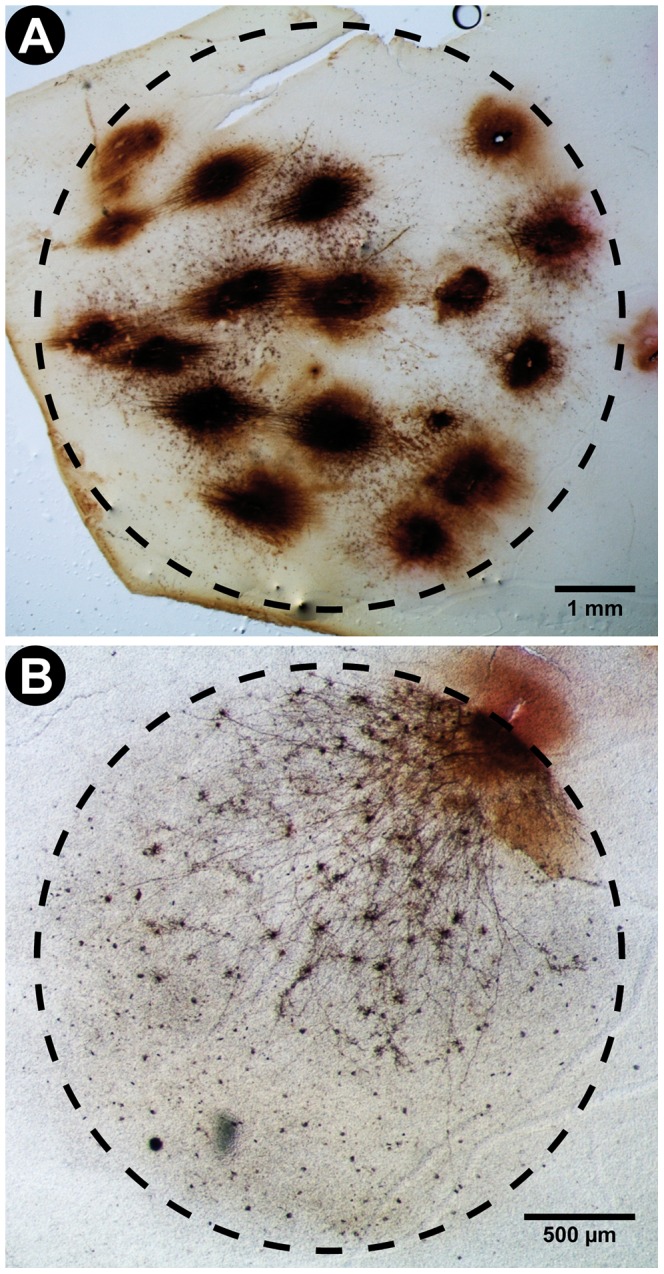
Representative photographs of the maximum illumination fields of the photooxidizer apparatus and of a conventional fluorescent microscope (Eclipse 80i microscope; Nikon, Japan). Illumination fields are defined by the region of the tissue were DAB was successfully photooxidized into an opaque polymer. The labeled structures include cell bodies, dendrites and axons of human retinal neurons. Observe that the geometry of the fields approximates that of a perfect circle. Also note that the illumination field area of the photooxidizer (42 mm^2^) is over 7.5 times larger than that of the microscope (5.5 mm^2^). This confirms the advantage of the new method for the processing large tissue samples. A: Very low magnification (1.6× objective lens) photographs of DAB-stained neurons in a human retina photooxidized using our new illumination method. The region of tissue that was successfully photooxidized can be fitted within a circle with a radius of approximately 3.66 mm and area of 42 mm^2^. Scale bar corresponds to 1 mm. B: Low magnification (4× objective lens) photographs of DAB-stained neurons in a human retina photooxidized using a conventional fluorescent microscope. The region of tissue that was successfully photooxidized can be fitted within a circle with a radius of approximately 1.32 mm and area of 5.5 mm^2^. Scale bar corresponds to 500 µm.

In order to ensure that the LED chamber heat sink was capable of successfully dissipating the heat generated by the illumination process, we filled the tissue chamber with deionized water (resistance of 18.6 MΩ) and measured the temperature variation within the chamber during four 15 minute bouts of continuous illumination, i.e. the same duration of illumination bouts used for controlled photooxidation reactions. Temperature measurements were carried out with a Fluke 51 K/J digital thermometer (Fluke Corporation, USA). We found that, starting from a mean room temperature of 25.55°C (SDM = 0.24), continuous LED illumination caused only a mean increase of 3.52°C (SDM = 0.17) within the chamber, with the maximum temperature not exceeding 29.3°C. This level of temperature increase clearly does not affect tissue integrity and did not affect photooxidation quality in our experiments (Figures 3–5, Table 1 and Figure S3), confirming that the heat dissipation system is functional and adequate for the desired purposes.

**Table 1:** Comparison between the new photooxidizer illumination method and conventional mercury arc lamp microscope photooxidation.

	Mercury lamp microscope*	Photooxidizer
**Staining quality**	Highly detailed staining of the whole cell	Highly detailed staining of the whole cell
**Illumination time**	2–3 hours	2–3 hours
**Illumination field**	5.5 mm^2^	42 mm^2^
**Estimated total cost**	≈US$500 (just for mercury lamp)	≈US$100 (for entire apparatus)
**Predicted lifetime**	2,500 hours	100,000 hours
**Predicted max. photooxidized area during lifetime**	≈4,583 mm^2^	1,400,000 mm^2^
**Estimated cost per hour**	US$0.20/hour	US$0.001/hour
**Estimated cost per area**	≈US$0.11/mm^2^	≈US$0.00007/mm^2^

* With a 10× objective lens and a 528–553 nm wavelength light filter.

### Animal manipulation procedures and collection of tissue samples

Samples of developing rat neocortex were obtained from Wistar rats at days P4–P6 of postnatal development. The rats were deeply anesthetized with ketamine (12 mg/kg; Vetanarcol, König, Brazil) and xylazine (90 mg/kg; Rompun, Bayer, Germany) and the level of anesthesia was assessed based on the animal's reaction to a toe pinch. The rats were then decapitated and their brains were removed and immersed in formaldehyde 4%. After at least two weeks of post-fixation, rat brains were mounted on a custom built vibratome and either tangential or coronal sections (70–100 µm thick) were obtained from area 17 of the visual cortex. Brain slices were then stored in formaldehyde 4% at 4°C until further procedures. Human retina samples were surgically removed during autopsy and stored in formaldehyde 4% at 4°C for at least two weeks. They did not receive any further treatment apart from dye labeling and histological processing.

### Histological processing and dye diffusion

Cells were labeled with the fluorescent, lipophilic carbocyanine dye DiI (1,1′, dioctadecyl-3,3,3′,3′-tetramethylindocarbocyanine perchlorate; Invitrogen, USA). As shown in Figure 2C, the emission spectrum of the photooxidizer apparatus encompasses the excitation wavelength of DiI (549 nm). A hypodermic needle was used to insert small beads of DiI into the parenchyma of each sample at several representative locations. The tissue was then stored in formaldehyde 4% at room temperature (≈23°C) for 1–4 weeks to allow for dye diffusion across the cellular membrane. After this period, DiI labeled cells were observed using fluorescent microscopy (Eclipse 80i microscope; Nikon, Japan) to confirm they were appropriately stained (Figures S1 and S2).

### Photooxidation protocol

Fluorescent marker photooxidation was performed using a modified version of the protocol by Sandell and Masland [4]. Briefly, the tissue samples were incubated in a chilled solution of 0.15% DAB (Sigma, USA) in 0.1 M Tris/HCl buffer (pH = 8.2). The tissue was held in place by the nylon mesh ring, directly on top of the LED's light beam. The DAB solution of the tissue chamber was changed every 15 minutes until the end of the protocol. The photooxidation process was periodically monitored using a low power light microscope (Standard 20; Zeiss, Germany) and lasted between 120–180 min. After photooxidation, tissues were washed overnight in 0.1 M Tris/HCl buffer and then mounted on a glass slide with Mowiol (Polysciences, USA).

In order to compare the efficiency of our method with currently standard illumination techniques, we performed control photooxidation experiments using a conventional broad spectrum mercury lamp as a light source. Microscope illumination was performed through a 528–553 nm light filter (G-2E/C; Nikon, Japan) and a 10× objective lens on an Eclipse 80i microscope (Nikon, Japan) with a mercury arc fluorescence lamp as the illumination source. The photooxidation protocol and histological procedures were exactly the same as those used for reactions in the photooxidizer, with the sole exception that the monitoring of staining quality was performed directly on the Eclipse 80i microscope.

### Image acquisition and comparison with conventional methods

Photooxidized tissue samples were visualized under bright field microscopy with an Eclipse 80i microscope and microphotographed at maximum resolution with a Nikon DS-Fi1c camera (Nikon, Japan). Photomicrographs were taken across various focal planes and then merged with the Helicon Focus® software (Helicon Soft, Ukraine) in order to allow the full visualization of neuronal processes across the depth axis. All images were adjusted for brightness and contrast across their entire span.

For the measurement of illumination field areas, successfully photooxidized tissue samples were photographed at very low magnifications with a M165 C stereomicroscope (Leica, Germany). The illumination field was defined as the area of the tissue sample that was selectively photooxidized during one procedure. Quantification of this area was performed using ImageJ software by defining a circle that encompassed the photooxidized cell bodies and neurites with the “elliptical selection” tool and then measuring its area with the “measure” command.

## Results and Discussion

### Staining quality

Our new method for fluorescent marker photooxidation yielded high quality and detailed images of neuronal morphology in nearly all processed tissue samples (Figures 3 and 4). Numerous cell bodies, dendritic arbors, dendritic appendages and axonal projections were labeled in all DiI-stained regions (Figures 3 and 4). Dendritic terminal clusters (Figure 4, Panels A–C) and axon terminal boutons (Figure 4, Panel D) could be clearly identified. Very similar results were obtained in control photooxidation reactions performed using the Eclipse 80i microscope, which confirms that the quality of photooxidation-based DAB staining performed with the new apparatus is on equal footing with current standard methodology (Table 1 and Figure S3). Likewise, the total illumination time necessary for high quality staining of neuronal cells was similar for both methods: 2 to 3 hours (Table 1). We were also capable of reliably visualizing the same set of cells under fluorescent (pre-photooxidation) and conventional (post-photooxidation) light microscopy without any obvious loss in staining quality (Figure S3).

In samples of developing rat neocortex, we retrieved images of DiI labeled Cajal-Retzius cells (Figure 3). The large unipolar horizontal-projecting dendrite of the Cajal-Retzius cell was clearly identified, as well as the dendritic side branches and spine-like appendages that are typical of this cell type (Figure 3, Panels A–B). The detailed visualization of these dendritic appendages highlights the acuity of the new method.

We were also able to successfully photooxidize DAB in fixed post-mortem human retinas. Most labeled neurons were identified as putative horizontal cells (Figure 4, Panels A–C). Photomicrographs of DAB-labeled cells revealed detailed images of neuronal cell bodies, dendritic arbors and axonal projections (Figure 4). Dendritic tufts, a typical characteristic of horizontal cells, were also clearly visualized (Figure 4, Panels A–C).

The produced results are well within the quality range for morphological reconstructions or neurite mapping studies [3], [15], [16]. Moreover, we confirmed that the quality of the images obtained with the new illumination method is at the same level as that obtained with a mercury lamp microscope, as both methods wielded highly detailed stainings of the entire cell and its processes (Figure S3 and Table 1). However, a few requirements must be followed to obtain a high quality labeling: a) the DAB solution should be filtered before use; preferentially with filters with a 40 µm pore diameter or less; b) the DAB solution should be periodically exchanged during the photooxidation process; we obtained optimal results by exchanging the solution every 15 minutes; c) materials used in the experiment must be regularly cleaned and always kept free of chemical or biological contaminants; d) the main area of interest for photooxidation should be kept in the center of the LED's light beam during the reaction.

### Methodological advantages of the new method

In addition to the already known advantages of photooxidation, including improved contrast and absence of photo-bleaching, our method makes the use of fluorescent microscopes unnecessary and allows for the simultaneous photooxidation of multiple labeled sites across large tissue samples, as it does not depend on the focal area of a microscope objective lens. These advantages make the method extremely cost and time efficient when compared with conventional photooxidation protocols, especially for large tissue samples, such as human retinas.

### Wide-field illumination for fluorescent photooxidation

One of the main advantages of the photooxidizer apparatus is its relatively wide light beam in comparison to microscope-based illumination methods. We measured the effective illumination field of both a conventional fluorescent microscope and the photooxidizer by quantifying the area of tissue that was successfully photooxidized within a single reaction session (2–3 hours). The criterion for a successful photooxidation was the same in both experiments, i.e., the imaging of high resolution DAB staining in cell bodies and neurites. We found that the illumination field in both cases can be approximated to a perfect circle (Figure 5). The region of tissue that was successfully photooxidized with the fluorescence microscope was fitted within a circle with approximately 1.32 mm of radius and an area of 5.5 mm^2^, while the region of tissue that was successfully processed with the photooxidizer was fitted within a circle with a radius of around 3.66 mm and area of 42 mm^2^ (Figure 5). Therefore, the new photooxidizer apparatus is capable of processing an area of tissue over 7.5 times larger than what would be possible with currently available methods, all within similar time frames and quality standards. This methodological advantage may be of high value for the detailed investigation of relatively large cellular and histological structures, such as neuronal projection fibers and individual axonal collaterals.

### Cost-efficiency of the new method

By eliminating the need for fluorescence microscopy, and consequently of mercury arc light bulbs, for the photooxidation reaction, the presented method provides a highly cost-efficient alternative to current protocols. This can be demonstrated by comparing the cost per hour of each methodological approach (summary in Table 1). Currently, high efficiency mercury arc lamps for fluorescence microscopy have a marketed average lifetime of approximately 2,500 hours and cost around US$500. This implies an estimated average cost of US$0.20/hour of lamp usage. Our apparatus, on the other hand, has a total cost of less than US$100, including the high power LED and all of its electronic and custom built components. Given that the high power LED we used has a 100,000 hour life expectancy, photooxidation with our method costs less than US$0.001/hour of usage (Table 1). In other words, we estimate the new method to be at least 200 times cheaper than current photooxidation techniques.

This cost difference becomes even more significant when one factors in the advantages of wide-field illumination (Table 1). Considering the measured illumination field areas in the conventional fluorescence microscope (5.5 mm^2^) and the photooxidizer (42 mm^2^), the life expectancy of high-end mercury arc lamps (2,500 hours) and high power LEDs (100,000 hour) and the fact that a successful photooxidation reaction takes a maximum of 3 hours with both methods, we estimate that the maximum possible area of tissue photooxidized with a mercury lamp during its entire lifetime is approximately 4,583 mm^2^, while the maximum possible area of tissue photooxidized with our system during the entire lifetime of a high power LED is 1,400,000 mm^2^. By dividing the cost of the mercury lamp and the photooxidizer apparatus by these area estimates, we predict a lifetime cost per area of processed tissue of around US$0.11/mm^2^ for conventional microscope-based techniques, while the cost per area of processed tissue with our new method would be around US$0.00007/mm^2^ (Table 1). Thus, by combining the benefits of high power LEDs and wide field illumination, our new method can potentially surpass the cost efficiency of currently available techniques by three orders of magnitude. We believe this massive decrease in cost might represent a major advantage for high throughput structural research in cell biology and neuroscience, such as high resolution mapping of brain structures.

Note that all estimates presented here considered the total cost of the apparatus; a comparison strictly between the use of mercury lamps and high power LEDs would have yielded an ever greater cost difference. It should also be noted that while many companies have recently presented microscope models that use high-power LEDs as an illumination source, this technology is far from being the standard in cell biology research. Furthermore, even with a LED-based light source, photooxidation efficiency with these microscope models would still be restricted by the relatively small illumination field of microscope optics. Thus, our new method offers immediate advantages that, to our knowledge, are currently unavailable to the general scientific community.

### Customizability and potential applications

Here we proved the concept of our new method by performing fluorescent photooxidation using intracellular DiI. However, the structure of the photooxidizer allows for a large degree of customization. By changing the high power LED for another with a different emission spectrum one can use this method to perform photooxidation reactions with any form of fluorescent marker, including commercial dyes and genetically encoded fluorescent proteins. Considering that LEDs have relatively broad emission spectra (Figure 2), all that is needed is for the investigator to use a LED whose spectrum contains the appropriate excitation wavelength for the marker of choice. For example, an appropriate high power LED for photooxidation using the yellow fluorophore DiO should have an emission spectrum that encompasses the 484 nm wavelength range. For Lucifer Yellow, the ideal emission spectrum would need to include a 428 nm wavelength. The same principle can be applied for genetically encoded markers: a good LED for photooxidation using EGFP would have an emission spectrum that encompasses the 488 nm wavelength range [13], while for the novel MiniSOG marker it would need to include the 448 nm wavelength range [14]. High power LEDs are cheap (approximately US$10) and easily replaceable within our apparatus schematics, both factors that highlight the customizability potential of the new approach.

Photooxidation techniques have been widely used in studies of neuronal tracing and of neurite morphology [1], [3], [8], [10], [17]. The use of retrograde or anterograde fluorochromes for neurite labeling is a classical approach for visualizing cell morphology and long range projections in nervous tissue. Fluorescent DAB photooxidation improves these methods by eliminating the risk of information loss by photobleaching and by providing images with improved visual contrast for manual or computer-assisted neuronal reconstructions [1], [3], [10], [17]. Another main advantage of the DAB products generated by fluorescent photooxidation is that they are electron-dense and osmiophilic, thus allowing for visualization under transmission electron microscopy [2], [3], [7], [13], [14], [18]. Previous studies have applied this principle to visualize specific organelles, cellular compartments, lipoproteins and proteins [2], [11], [12], [14], [18], [19]. In this context, our approach has a broad range of potential applications, from the study of general cellular morphology, including the reconstruction of neuronal processes and long range mapping of neural projections, to the analysis of cellular and molecular ultrastructure under electron microscopy.

## Conclusion

We developed and tested a new method for the photooxidation of fluorescent markers. Our novel LED-based approach is capable of producing high quality images of labeled neurons in rat and human tissue. This method allows the investigator to process large areas of tissue, does not rely on fluorescent or confocal microscopy and is at least 200 times cheaper and 7.5 times more efficient than current photooxidation protocols.

## Supporting Information

Figure S1 **Representative photomicrographs of DiI-filled neocortical neurons before the submission of the tissue samples to the photooxidation reaction.** Observe that the neuronal processes are well filled, a factor that is paramount for a good DAB staining in the subsequent photooxidation reaction. Scale bars correspond to 20 µm. A and B: Photomicrographs (40× objective lens) of neocortical neurons filled with DiI in samples of developing rat neocortex.
(TIF)

Figure S2 **Representative photomicrographs of DiI-filled human retinal neurons before and after the submission of the tissue samples to photooxidation reaction with our novel method.** Note that we were able to image the same group of cells under fluorescent microscopy (before photooxidation) and under light microscopy (after photooxidation). These images illustrate both the reliability of the method and the important advantage of photooxidation in general for studies of cellular morphology, given that the resolution for visualizing neuronal processes is greatly increased by this process. Colored arrows identify specific cells that are present in both panels. Scale bars correspond to 50 µm. A: Photomicrograph (20× objective lens) of neurons filled with DiI in samples of post-mortem human retina. B: Photomicrograph (20× objective lens) of the same neurons imaged in Panel A after submitting the tissue sample to photooxidation reaction with our novel apparatus.
(TIF)

Figure S3 **Representative photomicrographs of DAB-stained human retinal neurons photooxidized with our new illumination method and with a traditional mercury lamp-based technique.** Note that both illumination methods produce images of similar quality, i.e. they result in a highly detailed staining of the entire cell, including the dendrites and dendritic appendages. Scale bars correspond to 10 µm. A and B: Photomicrograph (100× objective lens) of DAB-stained human retinal neurons photooxidized with the novel photooxidizer apparatus. C and D: Photomicrograph (100× objective lens) of DAB-stained human retinal neurons photooxidized with a conventional fluorescent microscope.
(TIF)

File S1 **Detailed schematics for building the photooxidizer.**
(PDF)

Table S1 **Complete list of all commercial components of the photooxidizer apparatus.**
(DOCX)

Table S2 **Complete list of all custom built components of the photooxidizer apparatus.**
(DOCX)
