# Supplementary figures and images for: A New Low Cost Wide-Field Illumination Method for Photooxidation of Intracellular Fluorescent Markers
Source: PLoS One. 2013 Feb 18;8(2):e56512. doi: 10.1371/journal.pone.0056512 (PMC3575488; doi:10.1371/journal.pone.0056512)

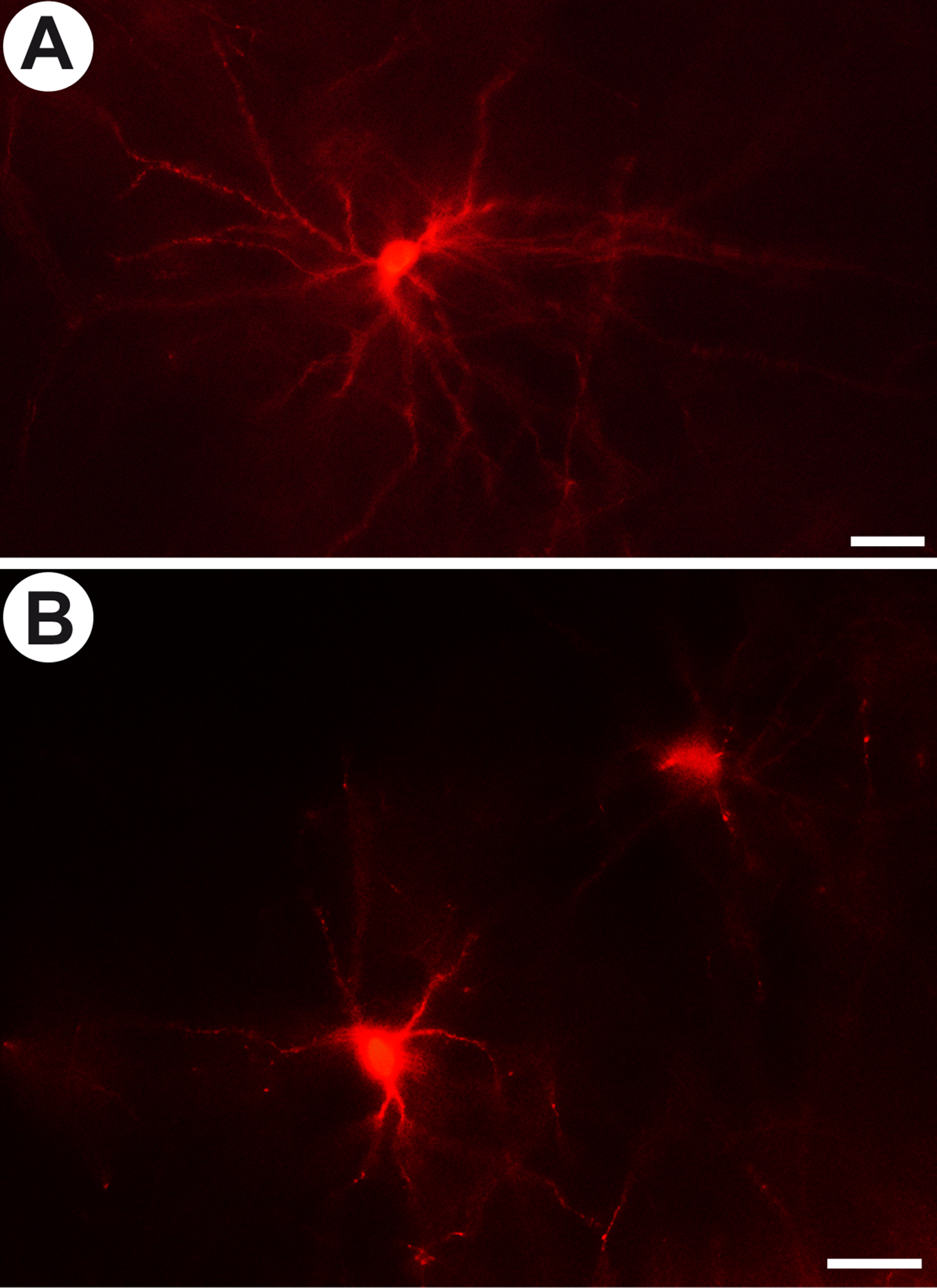

Supplement: Figure S1 — Representative photomicrographs of DiI-filled neocortical neurons before the submission of the tissue samples to the photooxidation reaction. Observe that the neuronal processes are well filled, a factor that is paramount for a good DAB staining in the subsequent photooxidation reaction. Scale bars correspond to 20 µm. A and B: Photomicrographs (40× objective lens) of neocortical neurons filled with DiI in samples of developing rat neocortex. (TIF) [file pone.0056512.s001.tif]

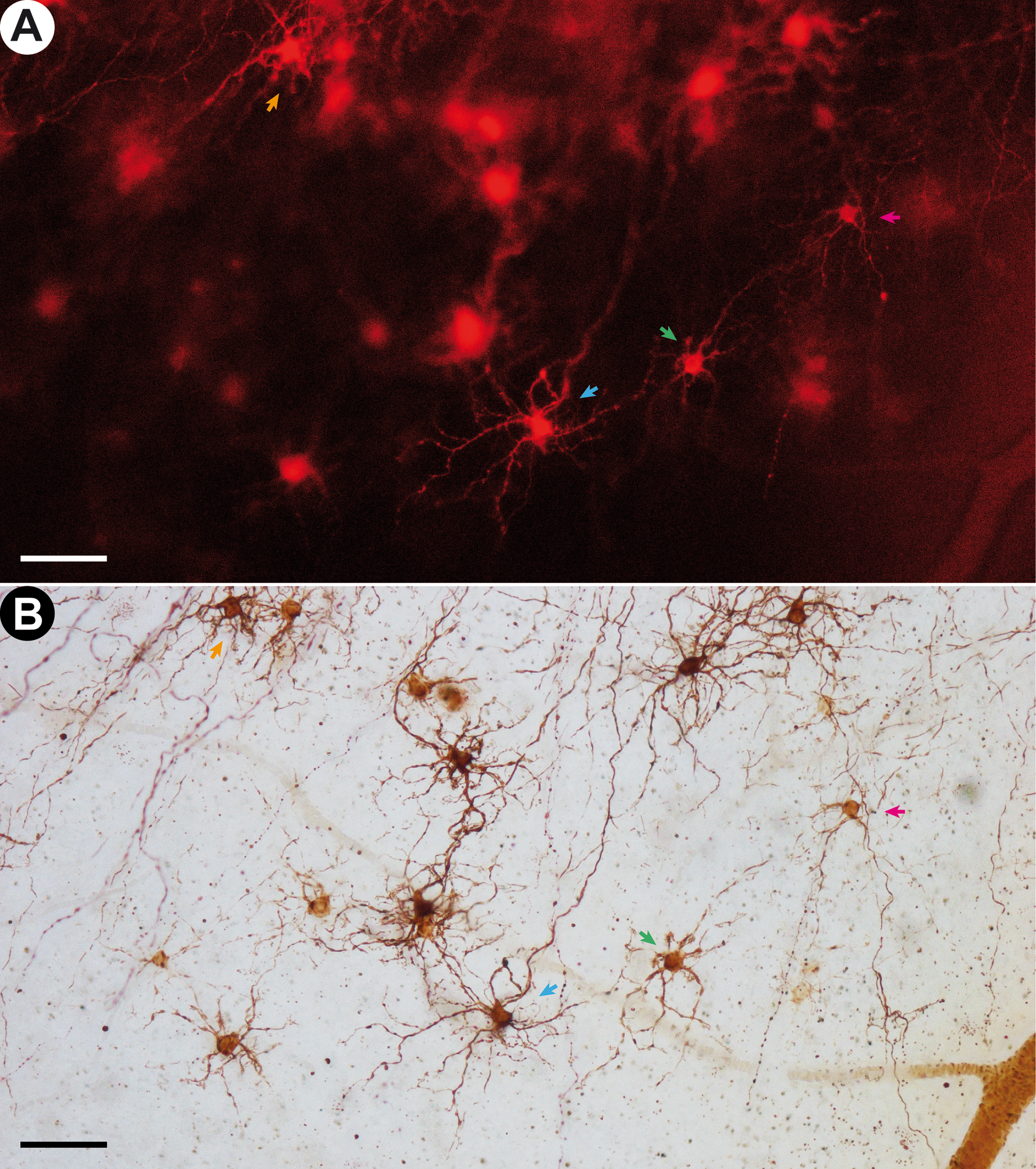

Supplement: Figure S2 — Representative photomicrographs of DiI-filled human retinal neurons before and after the submission of the tissue samples to photooxidation reaction with our novel method. Note that we were able to image the same group of cells under fluorescent microscopy (before photooxidation) and under light microscopy (after photooxidation). These images illustrate both the reliability of the method and the important advantage of photooxidation in general for studies of cellular morphology, given that the resolution for visualizing neuronal processes is greatly increased by this process. Colored arrows identify specific cells that are present in both panels. Scale bars correspond to 50 µm. A: Photomicrograph (20× objective lens) of neurons filled with DiI in samples of post-mortem human retina. B: Photomicrograph (20× objective lens) of the same neurons imaged in Panel A after submitting the tissue sample to photooxidation reaction with our novel apparatus. (TIF) [file pone.0056512.s002.tif]

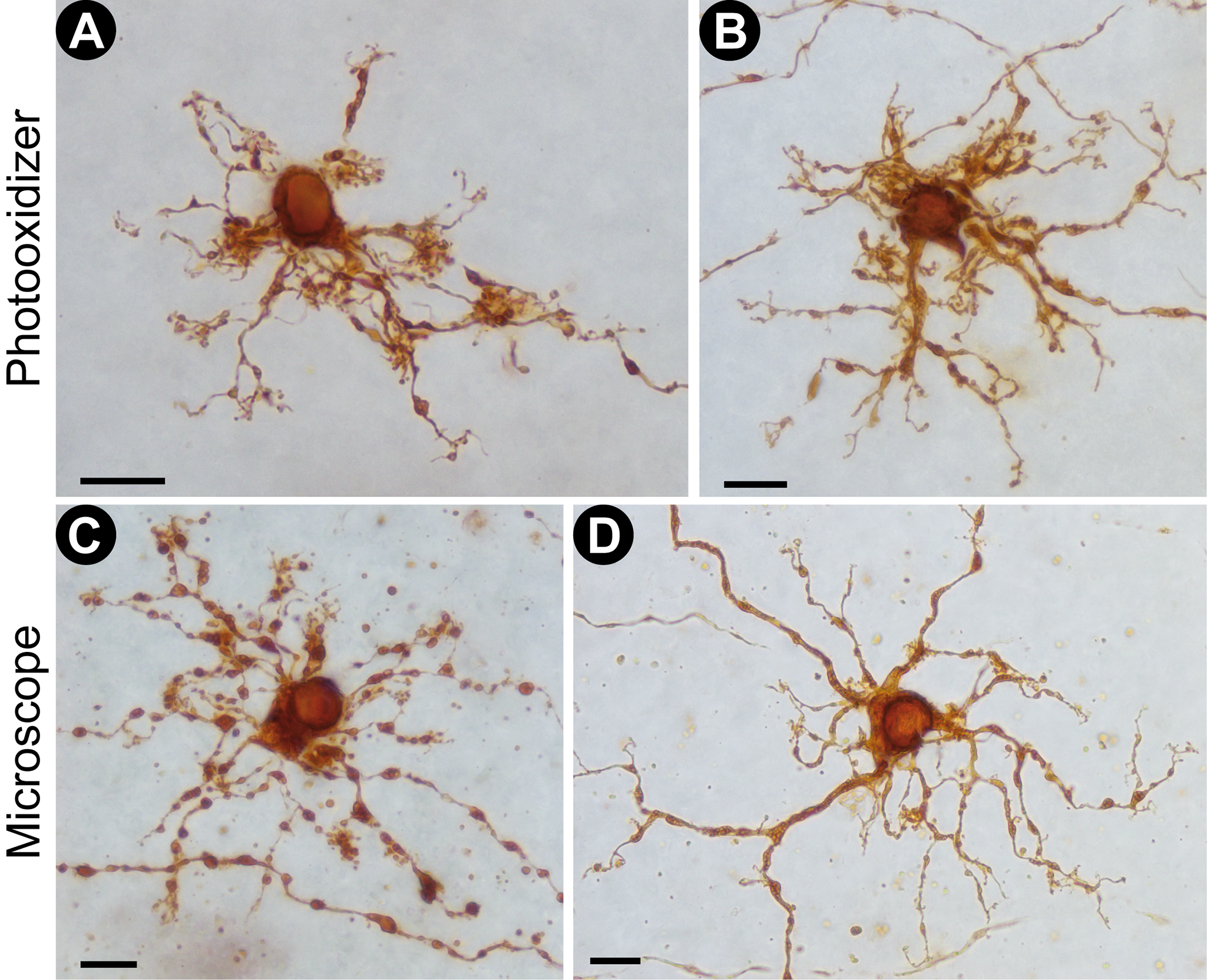

Supplement: Figure S3 — Representative photomicrographs of DAB-stained human retinal neurons photooxidized with our new illumination method and with a traditional mercury lamp-based technique. Note that both illumination methods produce images of similar quality, i.e. they result in a highly detailed staining of the entire cell, including the dendrites and dendritic appendages. Scale bars correspond to 10 µm. A and B: Photomicrograph (100× objective lens) of DAB-stained human retinal neurons photooxidized with the novel photooxidizer apparatus. C and D: Photomicrograph (100× objective lens) of DAB-stained human retinal neurons photooxidized with a conventional fluorescent microscope. (TIF) [file pone.0056512.s003.tif]
