# Supplementary material for: A New Low Cost Wide-Field Illumination Method for Photooxidation of Intracellular Fluorescent Markers
Source: PLoS One. 2013 Feb 18;8(2):e56512. doi: 10.1371/journal.pone.0056512 (PMC3575488; doi:10.1371/journal.pone.0056512)
Supplement: File S1 — Detailed schematics for building the photooxidizer. (PDF) [file pone.0056512.s004.pdf]

File S1: Detailed schematics for building the photooxidizer

Suplemental File to the article:

A new low cost method for photooxidation of intracellular fluorescent markers

by

Manoel da Silva Filho, Daniel Valle Vasconcelos Santos and Kauê Machado Costa

Printed circuit board (PCB) configuration and manufacture

For the construction of the PCB, copper tracing design was determined with a photolithographic subtractive method. In brief, the blank PCB sheet was coated with ultraviolet-sensitive positive photoresist (FASTPCB, Microtecnet Technology); the photolith design (represented below) was then drawn with opaque paint over the sheet. After exposure to ultraviolet light for 15 min, the PCB sheet was immersed in a solution of Na_2CO_3 (95 mM), removing the exposed photoresist coverage and leaving out the imprinted design. Copper etching was then performed using a solution of $FeCl_3$ (3 M) at 45 °C under periodic manual agitation for approximately 30 minutes. At the end of the procedure, the opaque paint covering the copper trace design was removed manually using a solution of NaOH (1.3 M) at 60 °C. After that, the PCB was cleaned, dried, drilled for screw placement and implanted with electronic components.

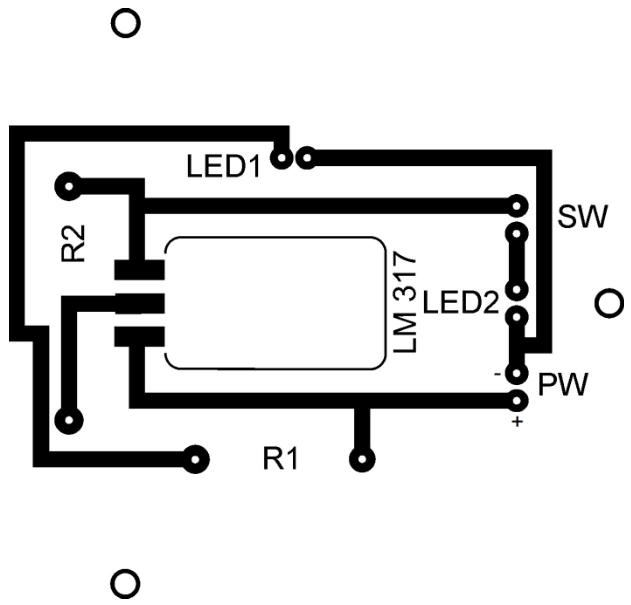

PCB photolith design. This design represents the pattern imprinted upon the photolith. R1: 470 Ω resistor. R2: 3.9 Ω resistor. LED1: Low power red LED. LED2: High power green LED. LM317: Voltage regulator. SW: On/off switch. PW: DC power input (9 Volts).

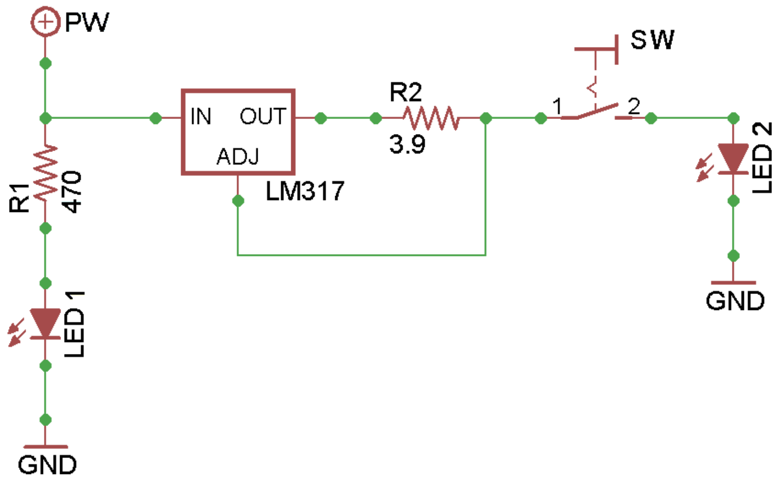

Electronic circuit schematics. R1: 470 Ω/1/4 W. R2: 3.9 Ω/1 W resistor. LED1: Low power red LED. LED2: High power green LED. LM317. SW: Switch on/off. PW: DC input.

Photooxidizer components

The following schematics represent the individual components of the photooxidizer apparatus, including internal grooves and entrances. All measures are in millimeters. The symbol "Ø" indicates measures of diameters.

Photooxidizer case

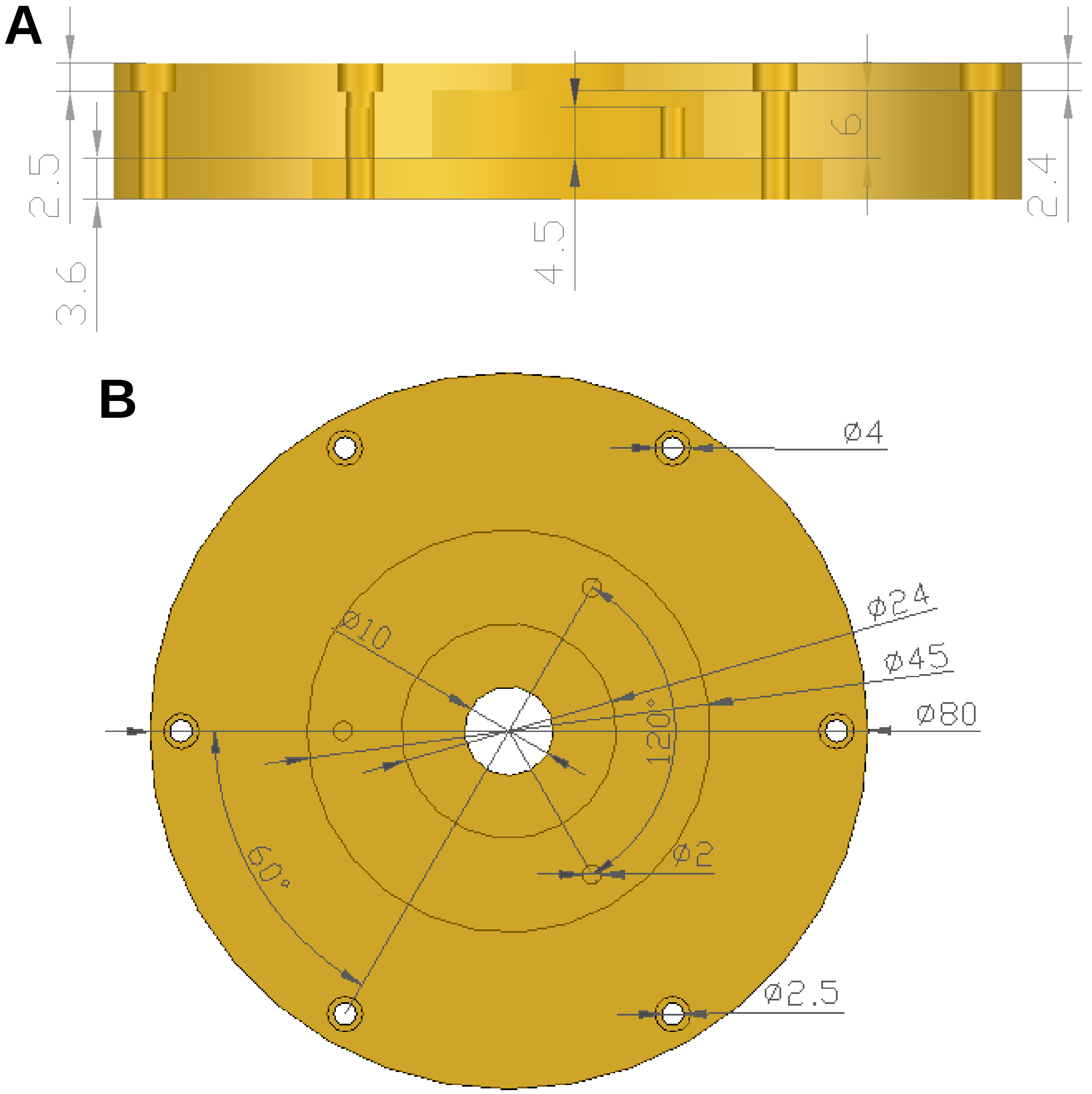

Component #1: top part of acrylic case. This component was built from a 12 mm thick acrylic sheet. The piece was shaped and grooved using an automated lathe. The pattern of grooves and recesses were designed to accommodate the LED chamber and to be aligned to the remaining parts of the external case. **A:** lateral view of the piece, with grooves and recesses indicated in an "X-ray" view. **B:** upper view of the component, with the diameter of the grooves and recesses indicated in grey. Six 2.5 mm diameter holes were made arranged in a 60° pattern, in order to screw the piece onto the other components of the photooxidizer. Each hole was surrounded by a 2.4 mm deep groove for accommodating the screw heads. Three 2.5 mm diameter holes were made arranged in a 120° pattern for screwing the LED chamber to the piece.

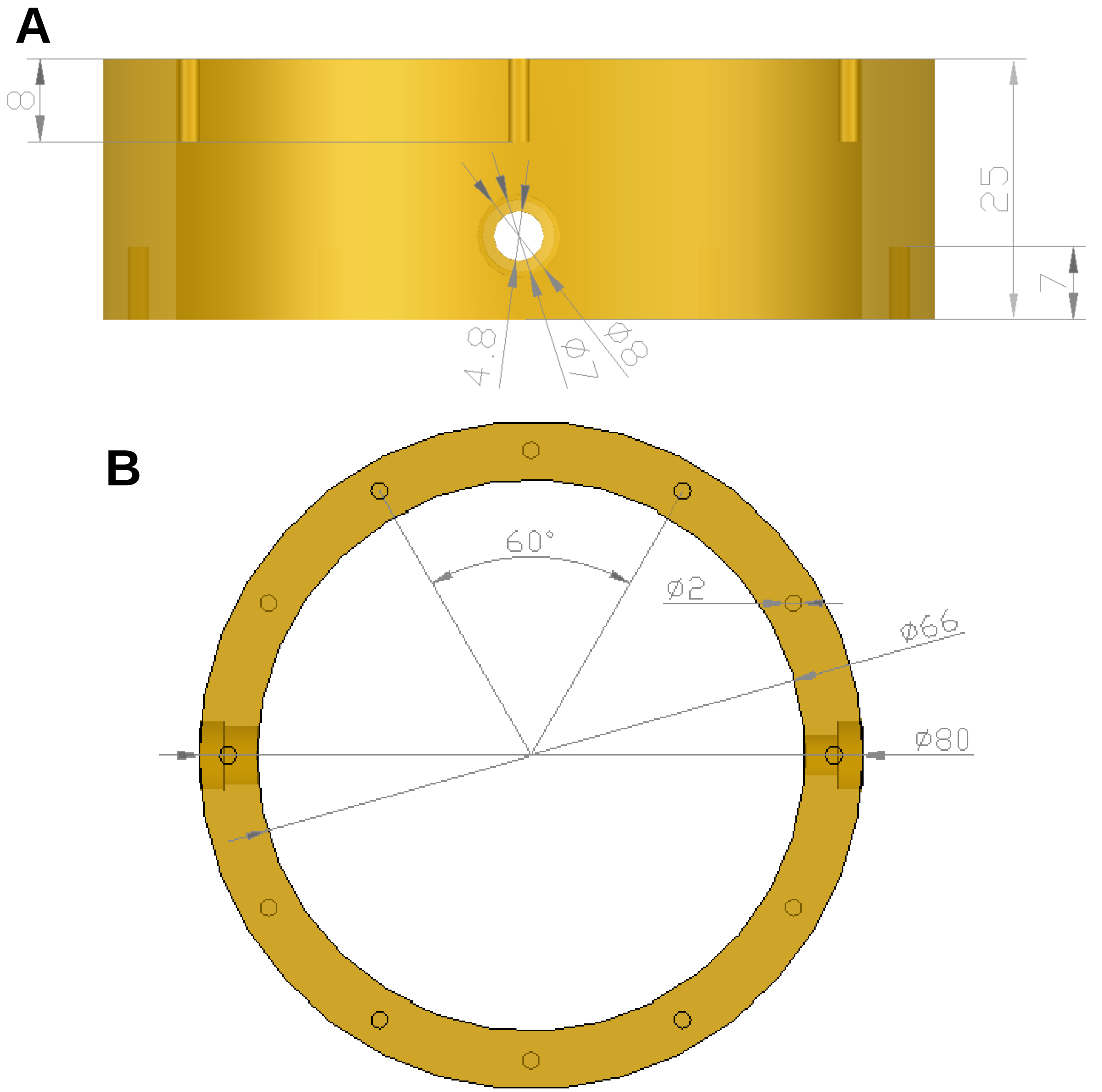

Component #2: middle part of acrylic case. This component was built from a 25 mm thick acrylic sheet. The piece was shaped and grooved using an automated lathe. The pattern of grooves and recesses were designed to accommodate the power feed connector and the on/off switch, as well as to be aligned to the remaining parts of the external case. **A:** lateral view of the piece, with grooves and recesses indicated in an "X-ray" view. **B:** upper view of the component, with the diameter of the grooves and recesses indicated in grey. Twelve 2 mm diameter holes were made arranged in a 30º pattern, in order to screw the piece onto the other components of the photooxidizer. Two simmetricallly opposite holes were drilled on the lateral sides of the piece in order to accommodate the on/off switch and the power feed connector.

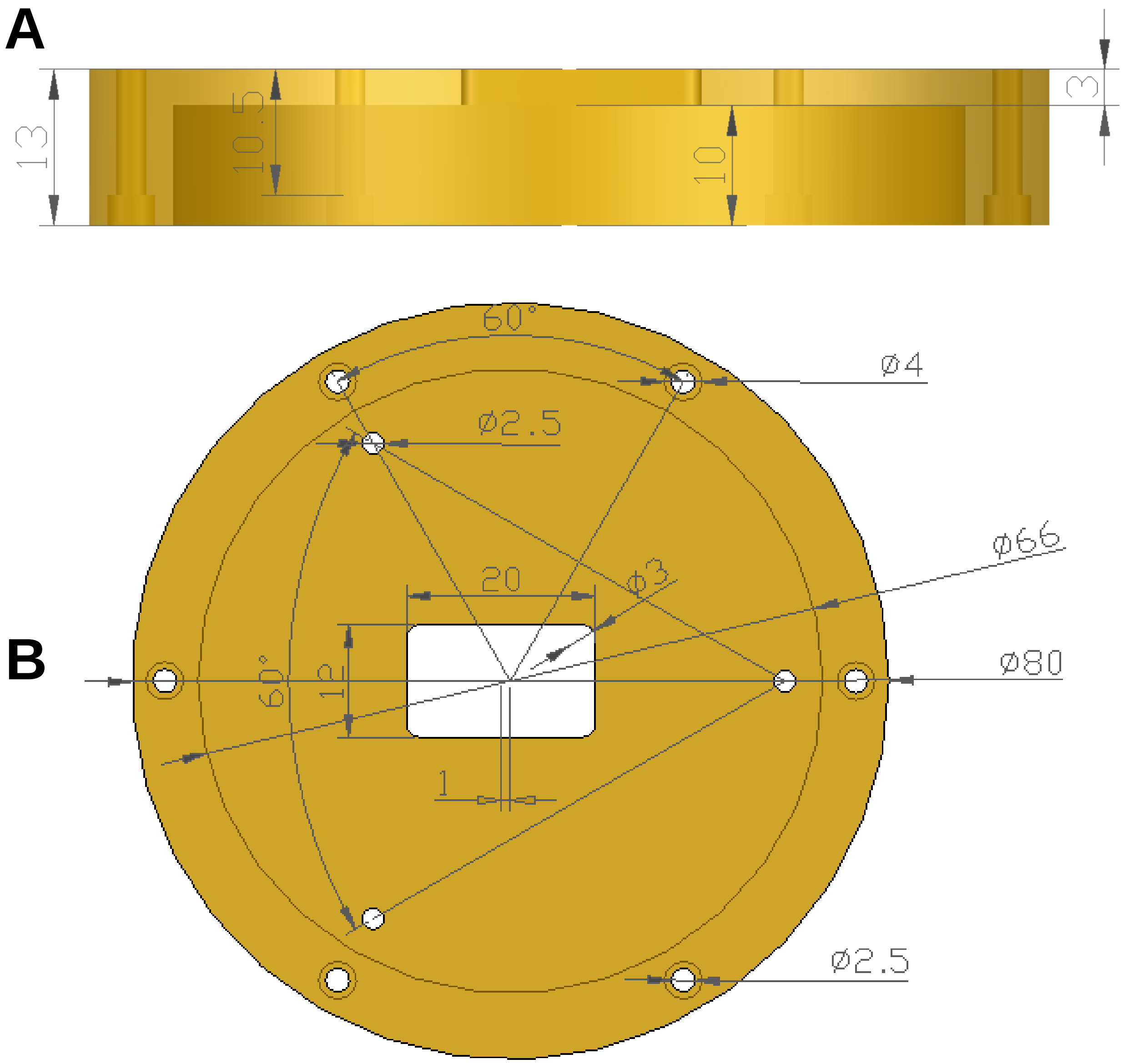

Component #3: bottom part of acrylic case. This component was built from a 13 mm thick acrylic sheet. The piece was shaped and grooved using an automated lathe. The pattern of grooves and recesses were designed to accommodate the PCB heat sink and to be aligned to the remaining parts of the external case. **A:** lateral view of the piece, with grooves and recesses indicated in an "X-ray" view. **B:** upper view of the component, with the diameter of the grooves and recesses indicated in grey. Six 2.5 mm diameter holes were made arranged in a 60º pattern, in order to screw the piece onto the other components of the photooxidizer. Each hole was surrounded by a 2.4 mm deep groove for accommodating the screw heads. Three 2.5 mm diameter holes were made arranged in a 120º pattern for screwing the PCB heat sink to the piece. A rectangular section (12 x 20 mm) of the acrylic sheet was also removed to accommodate the heat sink.

LED chamber and high power LED

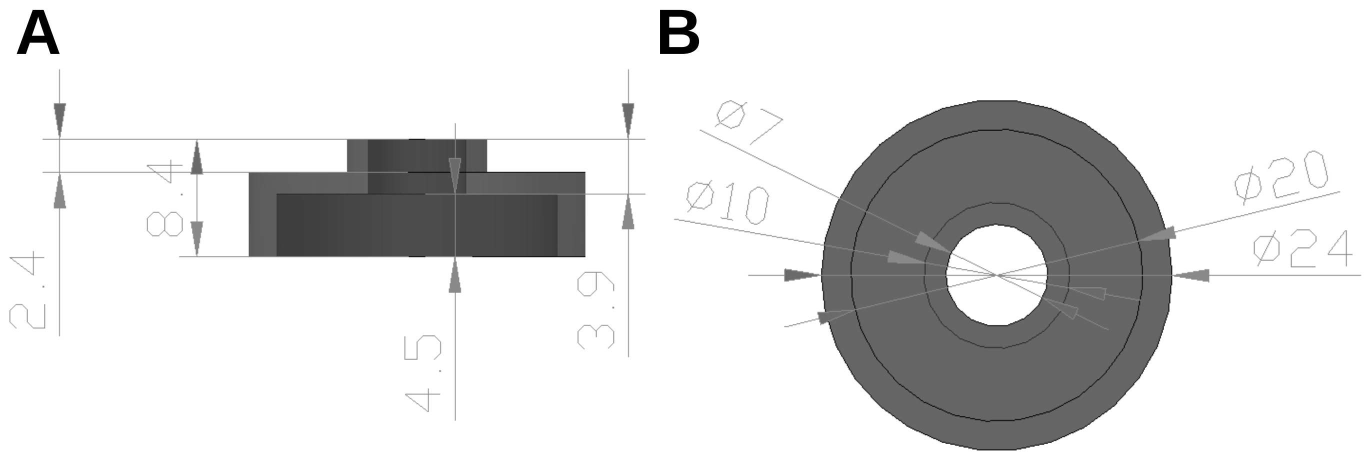

Component #4: aluminum LED chamber case. This component was built from a 24 x 8.4 mm aluminum cillinder.The piece was shaped, drilled and grooved using an automated lathe. The pattern of grooves and recesses were designed to accommodate the high power LED, the isolating Teflon® ring and to be aligned with the LED chamber heat sink. **A:** lateral view of the piece, with grooves and recesses indicated in an "X-ray" view. **B:** upper view of the component, with the diameter of the grooves and recesses indicated in grey.

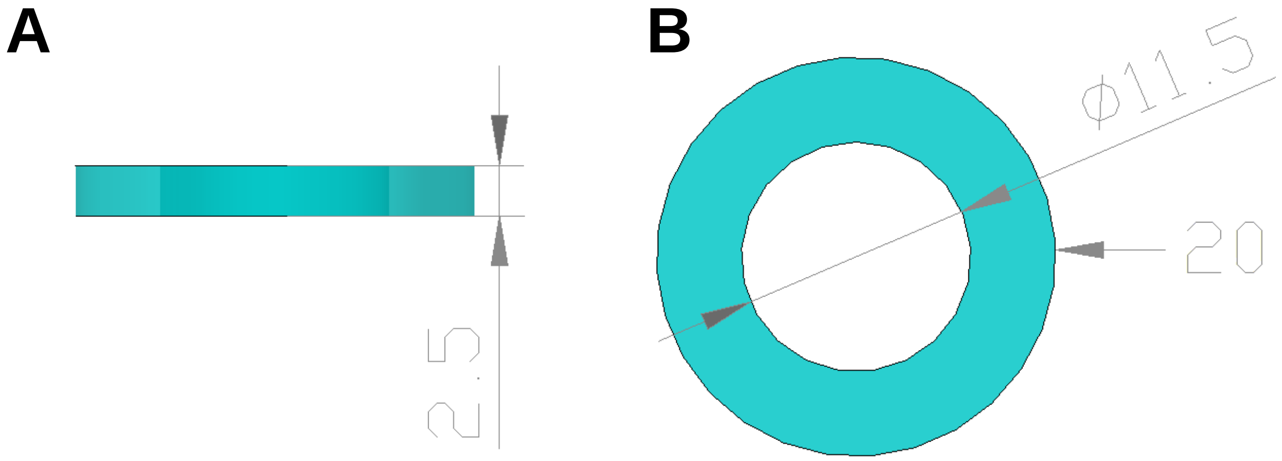

Component #5: Teflon® ring. This component was built from a Teflon® block that was shaped and drilled using an automated lathe to form a ring capable of surrowding the high power LED and fitting into the aluminum LED chamber.

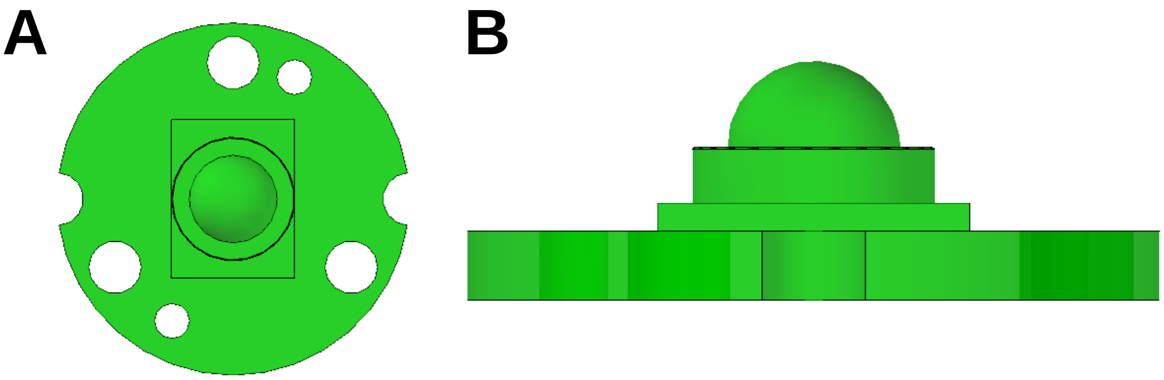

Component #6: High power LED. This component is commercially available and is manufactured by the company Cree (USA). The reference code for this component is XR7090GR. **A:** upper view of the piece. **B:** lateral view of the component.

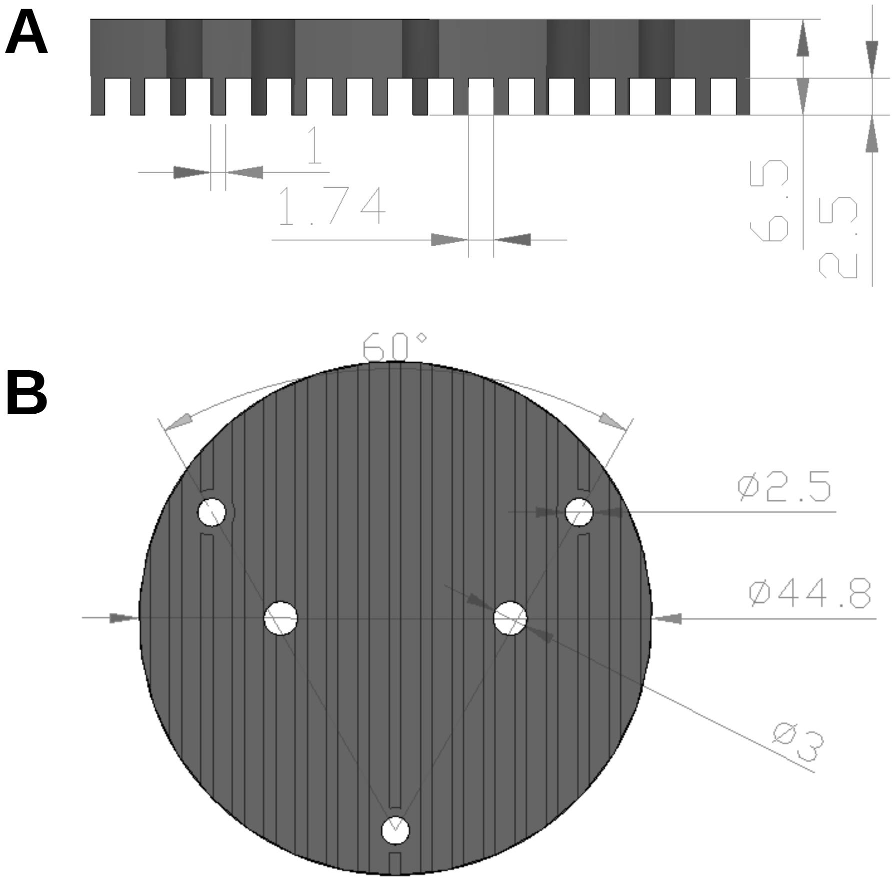

Component #7: aluminum LED chamber heat sink. This component was built from an aluminum sheet that was shaped, drilled and grooved using an automated lathe. The pattern of grooves and recesses were designed to efficiently dissipate heat across its surface, as well as to be aligned with the other LED chamber components. **A:** lateral view of the piece. **B:** upper view of the component, with the diameter of the grooves, holes and recesses indicated in grey.

Circuit, PCB and PCB heat sink

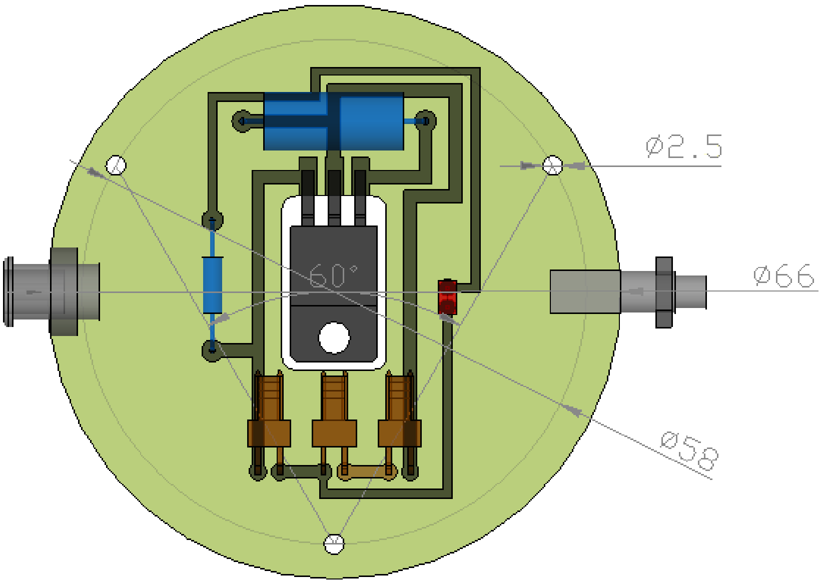

Component #8: Electronic circuit. This component was built according to the instructions detailed in the section "Printed circuit board (PCB) configuration and manufacture". The PCB was drilled with three holes in a 120º pattern, so as to be screwed to other components of the photooxidizer. All electronic components were soldered to the PCB before the final assembly process, with the exception of the on/off switch and the power feed connector, that were implanted only after the middle section of the acrylic case was in place.

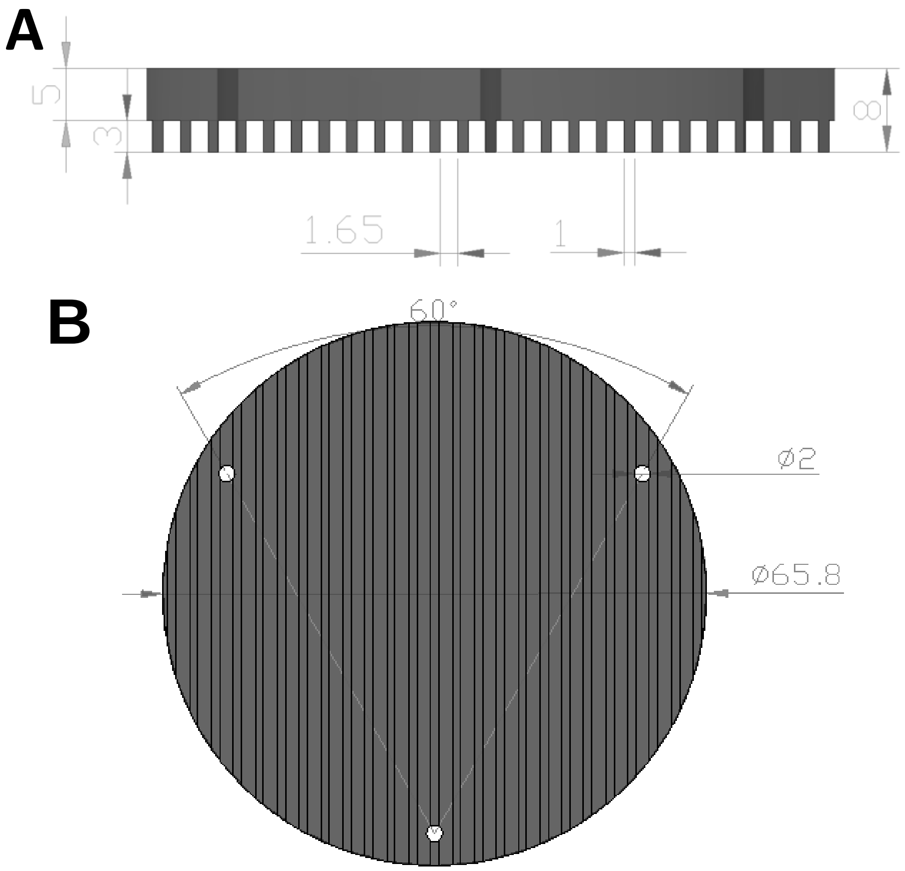

Component #9: PCB heat sink. This component was built from an aluminum sheet that was shaped, drilled and grooved using an automated lathe. The pattern of grooves and recesses were designed to efficiently dissipate heat across its surface, as well as to be aligned with acrylic case. **A:** lateral view of the piece. **B:** upper view of the component, with the diameter of the grooves, holes and recesses indicated in grey.

Screws

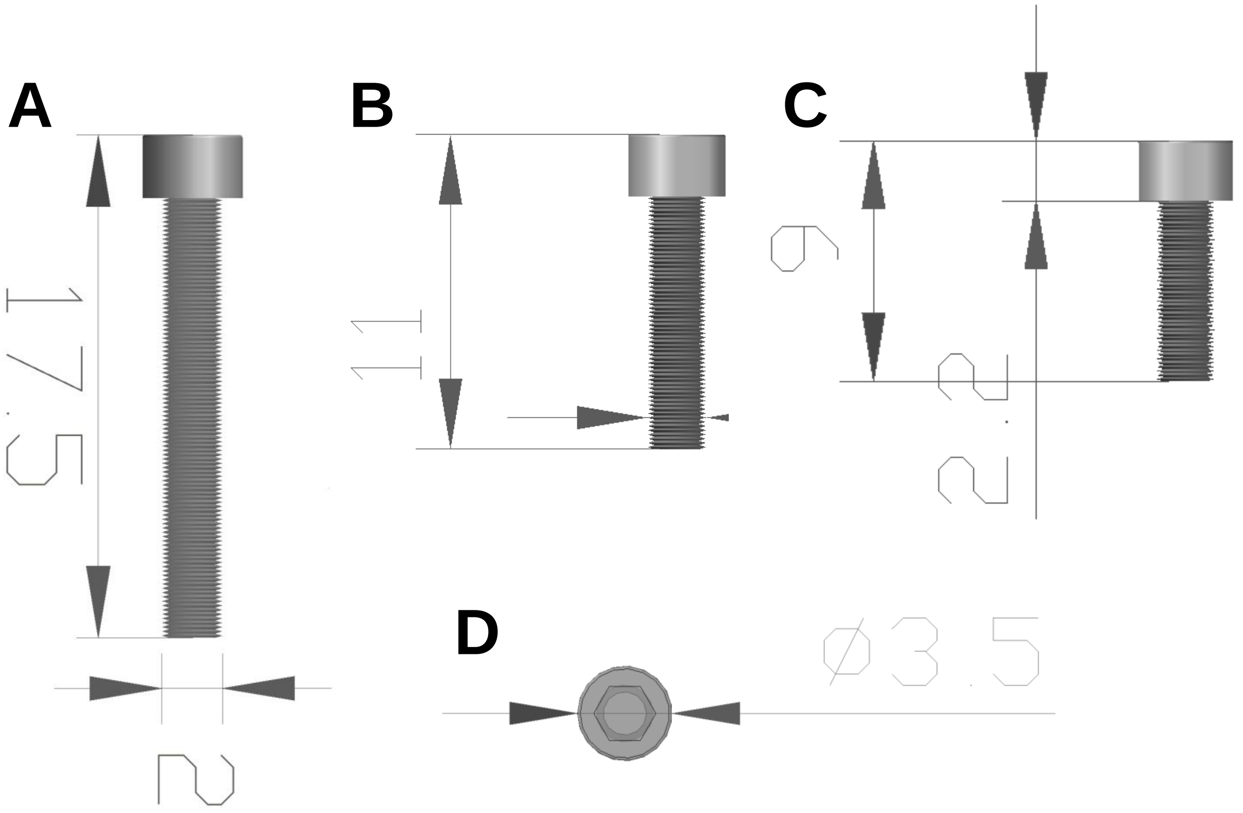

Component #10: Socket head screws. These components were acquired from the company Small Parts (USA). The reference code for this component is B005A0NVHE. All screws were originally 1 inch long and were sawed off to the size represented in the figure panels. **A:** lateral view of the 17.5 mm screw used to screw together the acrylic case. **B:** lateral view of the 11 mm screw used to screw the PCB electronic circuit and the PCB heat sink. **C:** lateral view of the 9 mm screw used to screw together the LED chamber. D: upper view of the socked head of all used screws.

Photooxidizer assembly and schematics

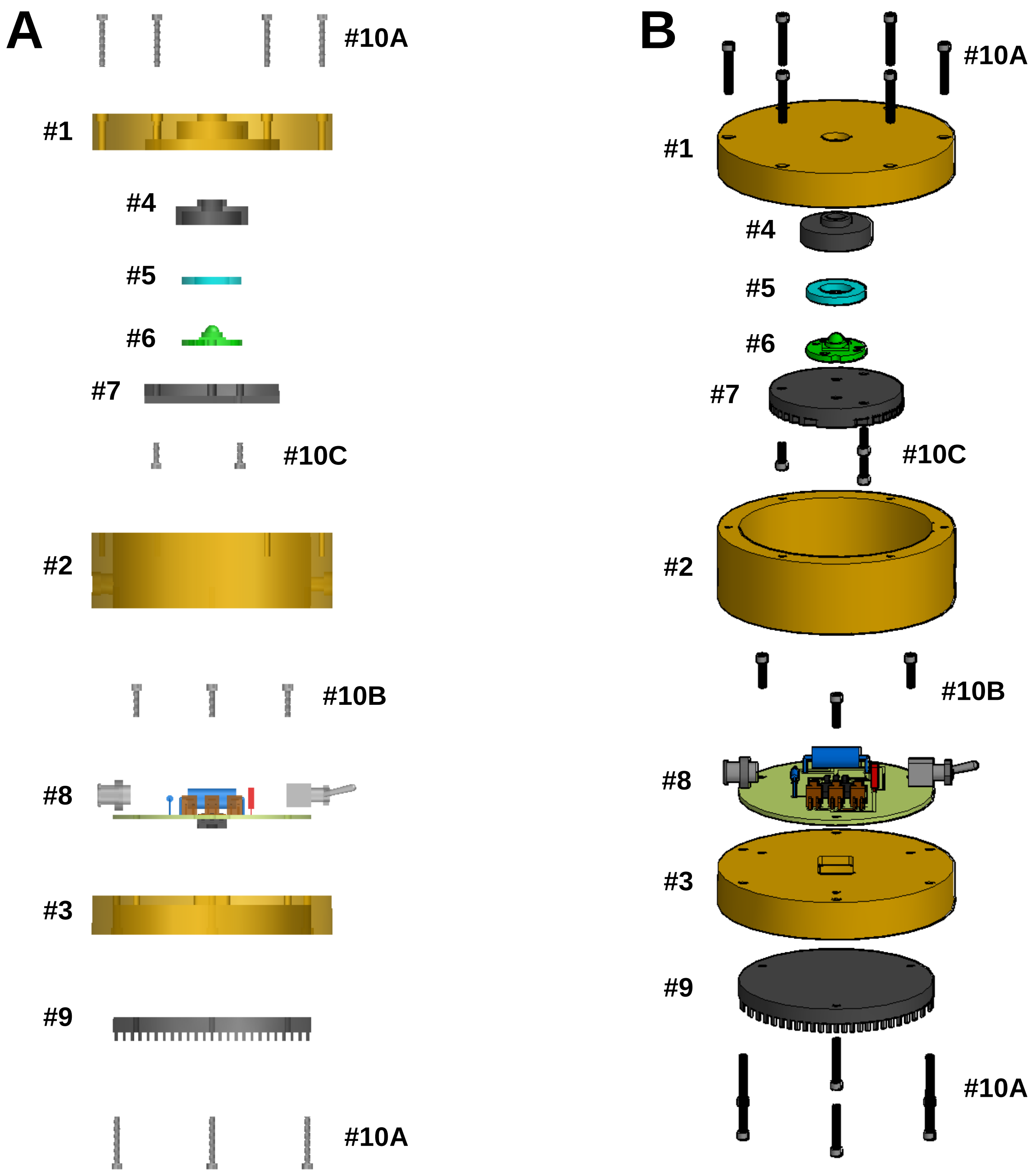

Exploded view of the photooxidizer. Schematics of the components of the photooxidizer and how they should be assembled. Note that the electronic components and connection wires should be soldered before the encasing of the internal components, with the exception of the on/off switch and the power feed connector, which must be inserted through the middle part of the acrylic case (component #2) before being connected to the PCB (component #8). Components are ordered and referenced according to the schematic presented in the section "Photooxidizer components". **A:** lateral view of the photooxidizer components, with grooves, holes and recesses displayed in an "X-ray" type view. **B:** perspective view of the photooxidizer components. **Legend:** #1: top part of acrylic case. #2: middle part of acrylic case. #3: bottom part of acrylic case. #4: LED chamber case. #5: Teflon® ring. #6: High power LED. #7: LED chamber heat sink. #8: PCB. #9: PCB heat sink. #10A: 17.5 mm long socket head screws. #10B: 111 mm long socket head screws. #10C: 9 mm long socket head screws.

Tissue chamber

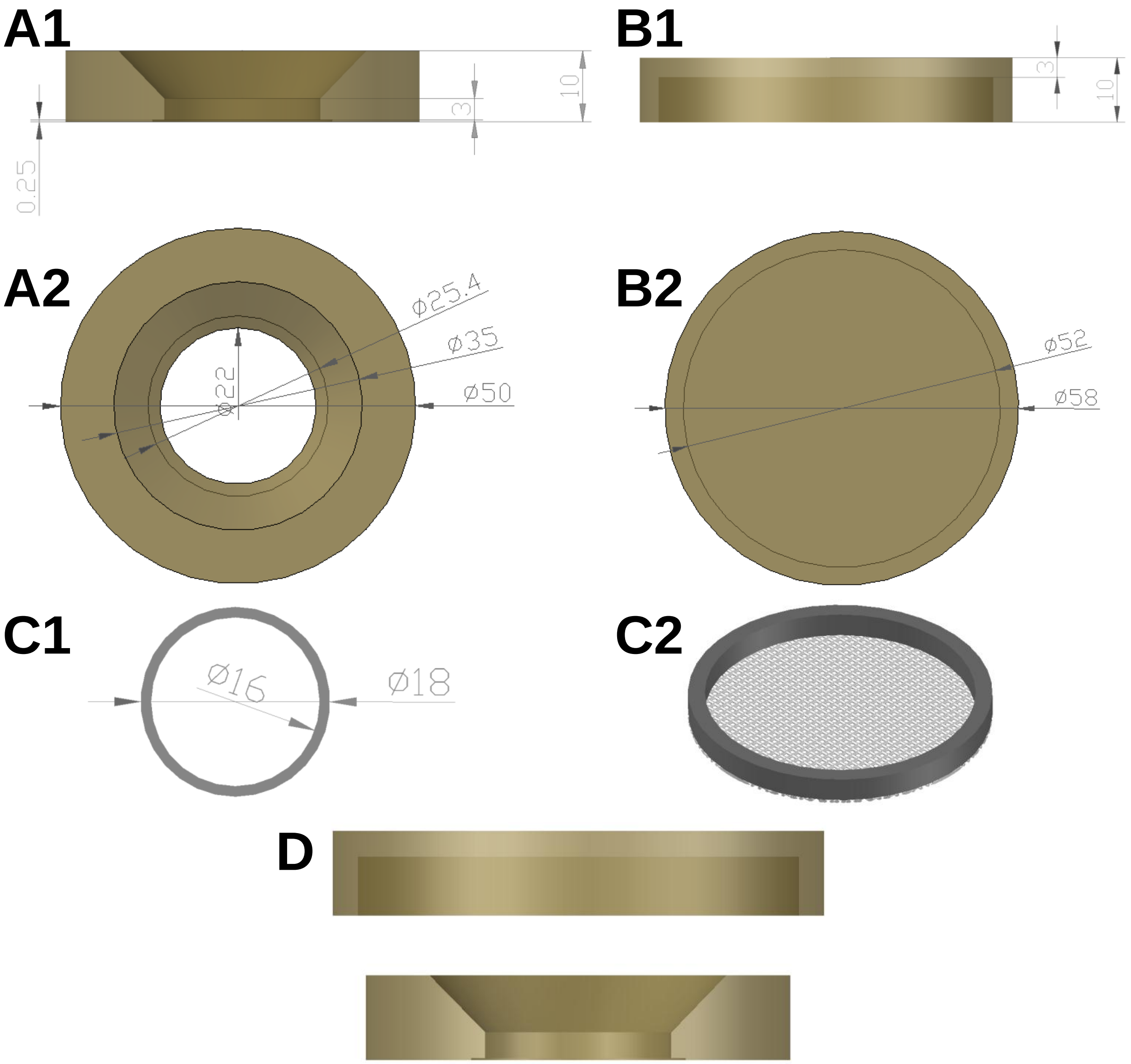

Tissue chamber schematics. The tissue chamber holds the samples during the photooxidation process. It is designed to keep the tissue in place both during the light induced reaction and during the periodic monitoring under a conventional microscope. The lower and upper parts of this component were built from an acrylic sheet that was shaped and drilled using an automated lathe. The ring that keeps the tissue samples in place was built from an aluminum sheet and a nylon mesh. A transparent glass microscope coverslip was glued under the lower piece using silicon glue. **A1:** lateral view of the lower piece, with grooves and recesses displayed in an “X-ray” view. Note the conical recess that surrounds the central hole. It allows for the better visualization of the tissue sample under a conventional microscope during. **A2:** upper view of the lower piece, with the diameter of the grooves, holes and recesses indicated in grey. **B1:** lateral view of the upper piece, or lid, of the chamber. Grooves and recesses are displayed in an “X-ray” view. **B2:** upper view of the lid, with the diameter of the grooves, holes and recesses indicated in grey. **C1:** Upper view of the stainless steel ring. **C2:** perspective view of the stainless steel ring and the nylon mesh. Then nylon mesh was glued to the stainless steel ring using cyanoacrylate glue. **D:** exploded view of the tissue chamber, with the grooves and recesses displayed in an “X-ray” type view.
