# Supplementary material for: A New Low Cost Wide-Field Illumination Method for Photooxidation of Intracellular Fluorescent Markers
Source: PLoS One. 2013 Feb 18;8(2):e56512. doi: 10.1371/journal.pone.0056512 (PMC3575488; doi:10.1371/journal.pone.0056512)
Supplement: Table S1 — Complete list of all commercial components of the photooxidizer apparatus. (DOCX) [file pone.0056512.s005.docx]

Table S1. Commercial components of the photooxidizer apparatus.

| Component | Description | Manufacturer | Reference |
| --- | --- | --- | --- |
| Voltage regulator | Microchip that controls the energy feed to the high power LED | Texas Instruments, USA | LM317 |
| High power LED | Strong LED for inducing fluorophore photoconversion | Cree, USA | XR7090GRN |
| Low power LED | Low intensity LED that signals if the PCB’s circuit is being fed | LightComp LED Corp, USA | 250AUR4G |
| On/Off switch | Small lever that controls the photooxidizer’s function | RadioShack, USA | 275-624 |
| Input/Output connectors | Connectors that mediate the input and output signals of the PCB’s circuit | Molex | MX-7395-02B  MX-6471-021 |
| 3.9 Ω resistor | Resistor that mediates current flow to the high power LED | RadioShack, USA | 1W3D9-10 |
| 470 Ω resistor | Resistor that mediates current flow to the low power LED | RadioShack, USA | 271-1317 |
| Stainless Steel Socket Head Cap Screws | Used for holding together the photooxidizer’s parts. Screws were sawed off to the sizes described in File S1. Hex Socket Drive, #1-72, 1" Length. | Small Parts, USA | B005A0NVHE |
| Power feed connector | Connects the photooxidizer to the external power source | Lemo, Switzerland | NIM-CAMAC 00.250 series |
| Power source | Regulates power feed to the photooxidizer | Hama, Germany | Universal-Schaltnetzgerät Eco 600 |
| Microscope glass slide | Bottom of the tissue chamber | Fisher Scientific, USA | 12-546-2 |
