# Supplementary material for: A New Low Cost Wide-Field Illumination Method for Photooxidation of Intracellular Fluorescent Markers
Source: PLoS One. 2013 Feb 18;8(2):e56512. doi: 10.1371/journal.pone.0056512 (PMC3575488; doi:10.1371/journal.pone.0056512)
Supplement: Table S2 — Complete list of all custom built components of the photooxidizer apparatus. (DOCX) [file pone.0056512.s006.docx]

Table S2. Custom built components of the photooxidizer apparatus.

| Component | Description | Materials |
| --- | --- | --- |
| Printed circuit board (PCB) | Circuit board designed for controlling the photooxidizer apparatus | FR-4 PCB |
| Photooxidizer case | Protective case for the internal circuitry of the apparatus | Transparent acrylic |
| Tissue chamber | External chamber for housing and positioning the tissue samples | Translucent acrylic and a microscope glass coverslip |
| Tissue chamber lid | Protective lid for the tissue chamber | Translucent acrylic |
| *Teflon^®^* ring | Piece that isolates the high power LED’s internal circuit | *Teflon^®^* |
| LED chamber | Chamber for housing the high power LED | Aluminum dowel |
| LED chamber heat sink | Grooved plate for dissipating excess heat | Aluminum plate |
| PCB heat sink | Grooved plate for dissipating excess heat | Aluminum plate |
